# Aberrant resting-state functional connectivity in medication-naïve generalized anxiety disorder: a whole-brain exploratory fMRI study

**DOI:** 10.3389/fpsyt.2025.1725066

**Published:** 2026-01-08

**Authors:** Yujing Jin, Tong Zhang, Wujianwen Zhai, Shuyi Liu, Yuxia Chen, Juhua Pan, Shijing Huang

**Affiliations:** Traditional Chinese Medicine Research and Development Center, Guang’anmen Hospital, Beijing, China

**Keywords:** default mode network, functional connectivity, functional magnetic resonance imaging, generalized anxiety disorder, posterior cingulate cortex, resting-state fMRI, supramarginal gyrus

## Abstract

**Background:**

Generalized Anxiety Disorder (GAD), characterized by excessive worry and somatic symptoms. Although neuroimaging studies have identified alterations in functional connectivity (FC), structural integrity, and neural activation in GAD, most include medicated or psychotherapy-treated patients, limiting insights into the neurobiology of the untreated state. This study investigated resting-state FC (rsFC) abnormalities in medication-naïve GAD patients using a whole-brain, data-driven approach.

**Methods:**

In this cross-sectional study, medication-naïve GAD patients (n = 85) and HCs (n = 82) underwent rs-fMRI at Guang’anmen Hospital on a Siemens 3.0T scanner. Data were analyzed using CONN toolbox (v22.v2407). After preprocessing, cluster-based rsFC was examined across 9, 453 connections in 138 ROIs (FSL Harvard-Oxford atlas, excl. cerebellum). Clusters correlated with HAMA scores; rsFC for 10 ROI pairs extracted via MATLAB; key ROIs seeded voxel-wise maps in SBC, controlling gender.

**Results:**

Significant group differences emerged in rsFC clusters, centered on connections between the posterior cingulate cortex (PCC) and right supramarginal gyrus (SMG). Compared to HCs, GAD patients exhibited hyper-connectivity in 5 connections and hypo-connectivity in 5 others within these clusters. Four connections showed positive correlations with HAMA scores.

**Limitations:**

The analysis of 9, 354 connections may have reduced statistical power, possibly obscuring additional relevant findings.

**Conclusion:**

This study demonstrates aberrant resting-state functional connectivity in medication-naïve GAD patients, particularly enhanced PCC-SMG rsFC correlated with anxiety severity, suggesting a potential role for interoceptive hypersensitivity in GAD pathophysiology. These findings support the hypothesis of SMG-driven vigilance engaging PCC and mPFC to perpetuate anxiety cycles, warranting future validation with direct interoceptive measures and highlighting neural targets for interventions.

## 1 Introduction

Generalized anxiety disorder (GAD) is defined by persistent, excessive, and uncontrollable worry—lasting for at least 6 months on most days—accompanied by somatic symptoms such as restlessness, fatigue, impaired concentration, irritability, muscle tension, and sleep disturbance (1). With a lifetime prevalence of approximately 6.2% (2), GAD imposes a substantial clinical and socioeconomic burden. Established treatments, such as cognitive behavioral therapy (CBT) (3), mindfulness-based stress reduction (MBSR) (4) and non-invasive brain stimulation (5), effectively reduce symptoms, yet the underlying neural mechanisms remain incompletely understood.

Neuroimaging evidence implicates dysregulated corticolimbic circuitry in the pathophysiology of GAD. Amygdala hyperactivity to threatening stimuli is a hallmark (6, 7), coupled with medial prefrontal cortex (mPFC) and anterior cingulate cortex (ACC) activation during worry (8). This is manifested by reduced functional connectivity (FC) between the amygdala and mPFC/ACC (9). Additional impairments occur in the ventrolateral prefrontal cortex (vlPFC), dorsolateral prefrontal cortex (dlPFC), posterior parietal regions, and amygdala (10), with altered amygdala-prefrontal FC underpinning excessive worry and autonomic dysregulation (11).

Hypothesis-driven analyses target the corticolimbic circuit—encompassing the amygdala, ACC, and prefrontal cortex (PFC)—where abnormal blood-oxygen-level-dependent (BOLD) responses predominate (12). Task-based functional magnetic resonance imaging (fMRI) reveals task-specific alterations in regions like the insula and hippocampus, but is limited by: small sample sizes constrain statistical power (13), and BOLD signal test-retest reliability is poor (14, 15).

Resting-state FC (rsFC) findings in GAD show heterogeneity, often attributable to seed region of interest (ROI) selection (16). Data-driven whole-brain methods address this by minimizing bias, such as through entropy measures like approximate entropy (ApEn) and sample entropy (SampEn) (17), or voxel-based FC strength-cerebral blood flow correlations (18). Integrating rsFC with structural MRI and graph theory further elucidates network and anatomical changes (19). A recent coordinate-based meta-analysis comparing rsFC in GAD and insomnia disorder highlights overlapping abnormalities in prefrontal and limbic regions, underscoring diagnostic overlap and the need for disorder-specific investigations (20).

Structural studies reveal impaired white matter integrity in emotion regulation tracts, including the uncinate fasciculus, inferior fronto-occipital fasciculus, and inferior longitudinal fasciculus (21). Gray matter reductions appear in the left superior temporal gyrus, with severity-linked changes in limbic areas like the lentiform nucleus and striatum (22). However, the ENIGMA Anxiety Working Group meta-analysis (n = 1112 GAD patients, 3282 healthy controls) detected no significant differences in cortical thickness, surface area, or subcortical volumes, indicating subtle or heterogeneous structural effects (23).

Research on first-episode, medication-naïve GAD patients reports negative correlations between left anterior insula-mPFC rsFC and somatic anxiety, though limited by small samples (e.g., n = 34 GAD, 30 controls) (24). The present study addresses these gaps with a larger medication-naïve sample to explore whole-brain rsFC abnormalities, employing a data-driven approach to overcome ROI biases and enhance understanding of untreated GAD neurobiology.

## 2 Materials and methods

### 2.1 Participants

This cross-sectional study was conducted at Guang’anmen Hospital, China Academy of Chinese Medical Sciences, between September 2022 and September 2024. A total of 94 medication-naïve patients with GAD and 91 healthy controls (HCs) were enrolled. All participants provided written informed consent in accordance with the Declaration of Helsinki. The study protocol was approved by the Institutional Ethics Committee (Approval No. 2022-081-KY).

### 2.2 Clinical assessment

GAD was diagnosed per the Diagnostic and Statistical Manual of Mental Disorders, Fifth Edition (DSM-5; American Psychiatric Association, 2013).

Inclusion criteria were: (1) age 18–69 years; (2) DSM-5 GAD diagnosis; (3) Hamilton Anxiety Rating Scale (HAMA) score ≥ 7; (4) right-handedness; (5) stable vital signs, clear consciousness, and adequate communication ability; (6) written informed consent.

Exclusion criteria were: (1) Hamilton Depression Rating Scale (HAMD-24) score ≥ 8; (2) anti-anxiety treatment in the past month; (3) history of alcoholism or substance dependence; (4) severe hepatic or renal insufficiency; (5) unstable vital signs; (6) pregnancy or lactation; (7) left-handedness; (8) MRI-incompatible implants (e.g., cardiac pacemakers, artificial heart valves, cochlear implants); (9) claustrophobia.

Both groups were evaluated for anxiety and depressive symptoms via the HAMA and HAMD - 24 assessments. We screened for psychiatric comorbidities and excluded individuals with recent anti-anxiety/antidepressant use, psychotherapy, or physical therapy.

Demographic and clinical data were analyzed in SPSS version 26.0. Normally distributed continuous variables with homogeneous variance were compared via independent-samples t-tests; non-normal variables used the Mann-Whitney U test. Categorical variables were assessed with chi-square tests. Significance was set at *p* < 0.05, with *p* < 0.01 denoting high significance.

### 2.3 MRI data acquisition

Scans were acquired at Guang’anmen Hospital using a Siemens Magnetom Skyra 3.0T scanner with a 20-channel phased-array head coil (Serial Number: 45617). Sequences included: (a) axial T1-weighted imaging (T1WI): repetition time (TR) = 2530 ms, echo time (TE) = 2.98 ms, matrix = 256 × 256, field of view (FOV) = 256 × 256 mm², slice thickness = 1 mm, slice gap = 1 mm, flip angle = 7°, voxel size = 1×1×1 mm³, 192 slices; (b) resting-state blood-oxygen-level-dependent (BOLD) imaging via gradient echo-echo planar imaging (GE-EPI): TR = 2000 ms, TE = 30 ms, matrix = 64 × 64, FOV = 224 × 224 mm², slice thickness = 3.5 mm, slice gap = 0.6 mm, flip angle = 90°, voxel size = 3.5 × 3.5 × 3.5 mm³, 36 slices.

### 2.4 Data preprocessing and analysis

Functional MRI (fMRI) data analyses were performed using CONN FC toolbox version 22.v2407 (25) and SPM12 version 25.25.01.02 (RRID: SCR_007037). Preprocessing steps included: 1. head motion correction. 2. slice-timing correction. 3. coregistration of structural and functional images. 4. spatial normalization. 5. scrubbing of timepoints exceeding 0.9 mm framewise displacement or > 5 standard deviations (26). 6. resampling to 2-mm isotropic voxels using the the IXI-549 template (default in CONN) tissue probability map template (27). 7. spatial smoothing with an 8-mm full-width at half-maximum (FWHM) Gaussian kernel. 8. bandpass filtering (0.01–0.08 Hz).

Denoising: Functional MRI (fMRI) data underwent standard denoising procedures involving regression of potential confounding effects characterized by white matter time series (5 CompCor noise components), cerebrospinal fluid time series (5 CompCor noise components), motion parameters with their first-order derivatives (12 factors), scrubbed scans (89 factors), session effects with their first-order derivatives (2 factors), and linear trends within each functional run (2 factors), followed by bandpass frequency filtering of BOLD time series at 0.008–0.09 Hz (28).

First-level analysis estimated region-of-interest-to-region-of-interest (ROI-to-ROI) connectivity matrices (RRC) and seed-based connectivity maps (SBC) based on 156 HCP-ICA networks (29) and Harvard-Oxford atlas ROIs (30). FC was quantified as Fisher-transformed bivariate correlations from weighted generalized linear models (weighted-GLM (31)), modeling BOLD time-series associations per seed-target pair. magnetization transient effects were addressed by weighting scans with a step function convolved with SPM’s canonical hemodynamic response function.

Group-level analysis used generalized linear models (GLMs), with first-level connectivity as the dependent variable and group as the independent variable. Voxel-wise tests applied multivariate parametric statistics with random effects and covariance estimates. Cluster-level inference relied on Gaussian random field theory (32), thresholded at voxel-level *p* < 0.01 and cluster-level *p-FDR* < 0.05 (33).

### 2.5 ROI-to-ROI analysis

We employed CONN’s default FSL Harvard-Oxford atlas (excluding cerebellum), yielding 9, 453 connections across 138 ROIs. rsFC was computed as Fisher z-transformed Pearson correlations from weighted-GLMs. Thresholds were connection-level *p* < 0.05 and cluster-level *p-FDR* < 0.05, covarying age and sex. Excessive motion (> 0.5 mm framewise displacement) was excluded via quality assurance outputs. SMG-PCC rsFC was specifically examined for clinical correlations.

### 2.6 rsFC correlation with clinical scores

HAMA assessed GAD severity. rsFC values for 10 predefined ROI pairs (including SMG and PCC) were extracted from CONN first-level Fisher z-transformed matrices using custom MATLAB scripts (n=185 participants). Variables met Shapiro-Wilk normality in SPSS 26.0. Pearson correlations with HAMA controlled for age and sex, with Bonferroni-corrected *p* < 0.05 (for 10 comparisons) to address multiplicity.

### 2.7 Seed-to-voxel analysis (SBC)

Key ROIs from RRC results served as seeds for voxel-wise maps in CONN’s SBC module. Despite seed selection challenges in whole-brain analyses (34), *post-hoc* exploration of identified ROIs facilitated rsFC pattern assessment (35). Group-level GLMs covaried age and sex. Inference used randomization/permutation statistics for contiguous voxel clusters (36), thresholded at voxel-level *p* < 0.01 and cluster-level *p-FDR* < 0.05 (CONN defaults).

## 3 Results

### 3.1 Demographic data and clinical characteristics

We initially recruited 185 participants (94 with GAD, 91 HCs). After excluding 6 GAD patients and 12 HCs for excessive head motion during fMRI (primarily due to scanner noise-induced discomfort, a common source of motion artifacts (37), the final sample consisted of 167 individuals (85 with GAD, 82 HCs).

The GAD group exhibited a mean HAMA score of 23.7 (SD = 3.7), indicative of moderate-to-severe anxiety, which was significantly higher than that of HCs (*p* < 0.001). Additionally, the GAD group showed significantly elevated Hamilton Depression Rating Scale (HAMD-24) scores compared to HCs (*p* < 0.001), likely due to anxiety-related items within the HAMD-24. This elevation did not affect the validity of subsequent statistical analyses.

Demographic and clinical characteristics are presented in **Table 1**. The GAD group had significantly higher HAMA scores (mean = 23.7, SD = 3.7) than HCs (*p* < 0.001), corresponding to moderate-to-severe anxiety levels. HAMD-24 scores were also elevated in the GAD group (*p* < 0.001), likely due to overlapping anxiety items, without impacting subsequent analyses.

**Table 1:** Demographic and clinical data of analyzed participants.

Variables	GAD (n = 85)	HC (n = 82)	*Z/x^2^*	*p*
	Mean ± SD	Mean ± SD		
Age (years)	44.0 ± 13.8	42.4 ± 13.4	0.752	0.512
Sex (male/female)	25/60	38/44	5.092	0.024
Duration (year)	1.2 ± 0.6			
HAMA	23.7 ± 3.7	5.0 ± 2.1	-11.18	< 0.001
HAMD-24	6.7 ± 2.0	1.9 ± 1.1	-10.74	< 0.001

Group differences were evaluated using *Mann-Whitney Test* for Age, HAMA and HAMD-24, and *chi-square test* for sex.

### 3.2 rsFC alterations detected in ROI-to-ROI analysis

**Figure 1:**
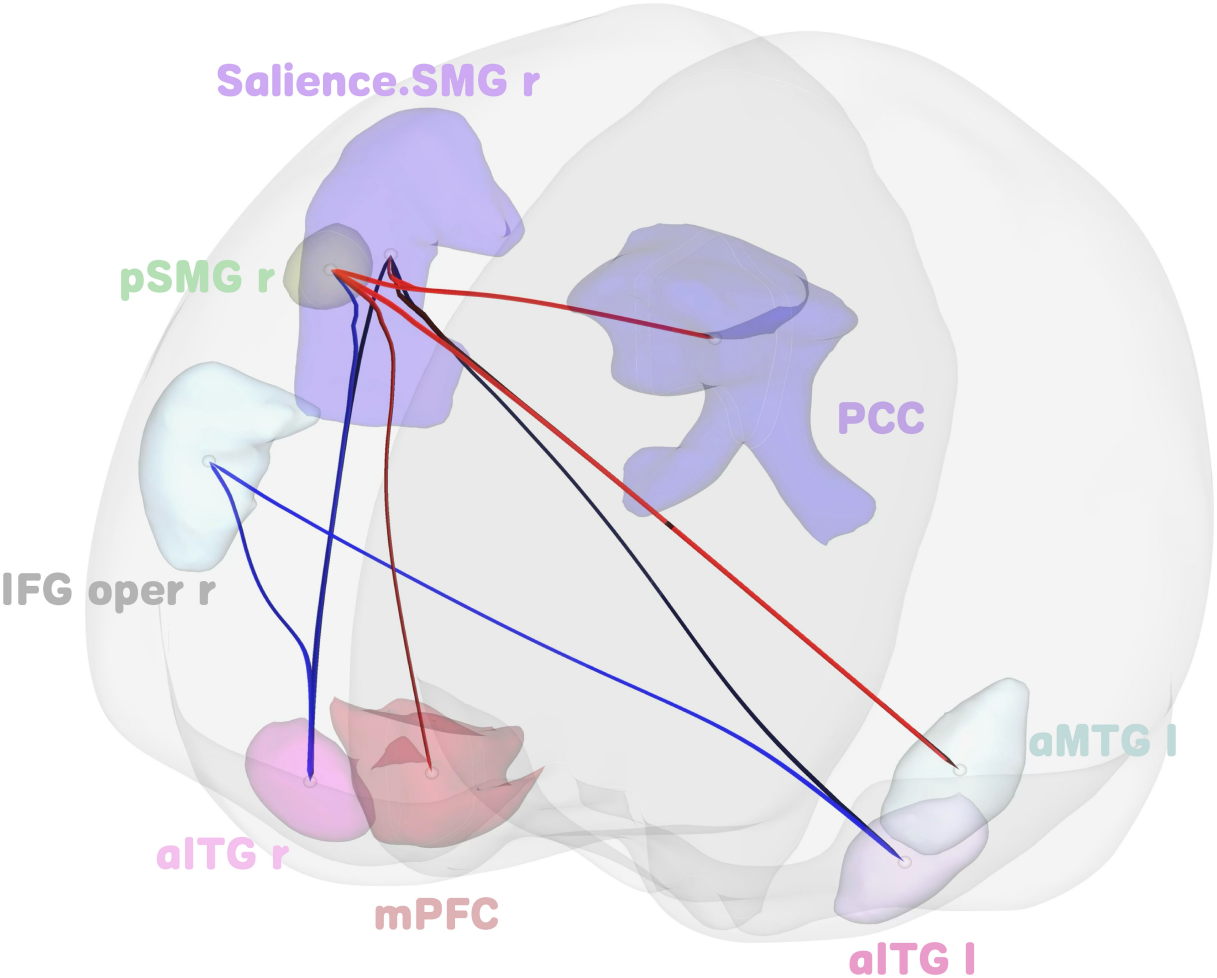
Three-dimensional rendering of brain regions exhibiting significant FC differences within clusters showing group differences between GAD patients and HCs. Salience.SMG r denotes the SMG r region within the salience network. Red lines represent hyper-connectivity (increased FC), while blue lines represent hypo-connectivity (decreased FC) relative to HCs.

Group differences in rsFC emerged at the cluster level between patients with GAD (n = 85) and HCs (n = 82) (F (4, 162)=6.6, *p* < 0.001, *p-FDR* = 0.013), involving two clusters: (1) right supramarginal gyrus (SMG) and right pars opercularis of the inferior frontal gyrus (IFG pars opercularis); (2) posterior cingulate cortex (PCC), medial prefrontal cortex (mPFC), left anterior inferior temporal gyrus (aITG), and left anterior middle temporal gyrus (aMTG). Regions with differential FC in these clusters are detailed in **Figure 1** and **Table 2**.

**Table 2:** Cluster and cluster-forming connections with significant difference between GAD and HC.

Cluster	*F(4, 162)*	*p*	*p-FDR*
	6.6	0.001 <	0.012764
Connections		*T*	*p*
pSMG r	aITG r	-2.72	0.007298
	aITG l	-2.59	0.010458
	aMTG l	2.51	0.013135
	PCC	2.22	0.027510
IFG oper r	aITG l	-2.26	0.024934
	aITG r	-2.19	0.029903
Salience.SMG r	PCC	2.34	0.020290
	aMTG l	2.29	0.023050
	mPFC	2.13	0.034414
	aITG r	-2.11	0.036113

F = MSB/MSW, representing the ratio of between-group variance (mean square between, MSB) to within-group variance (mean square within, MSW) in analysis of variance (ANOVA) for group-level inference. pSMG, posterior supramarginal gyrus; aITG, anterior inferior temporal gyrus; aMTG, anterior middle temporal gyrus; PCC, posterior cingulate cortex; IFG oper, inferior frontal gyrus, pars opercularis; Salience.SMG, supramarginal gyrus in the salience network; mPFC, medial prefrontal cortex; l, left; r, right.

### 3.3 Correlation between clinical scores and rsFC

**Figure 2:**
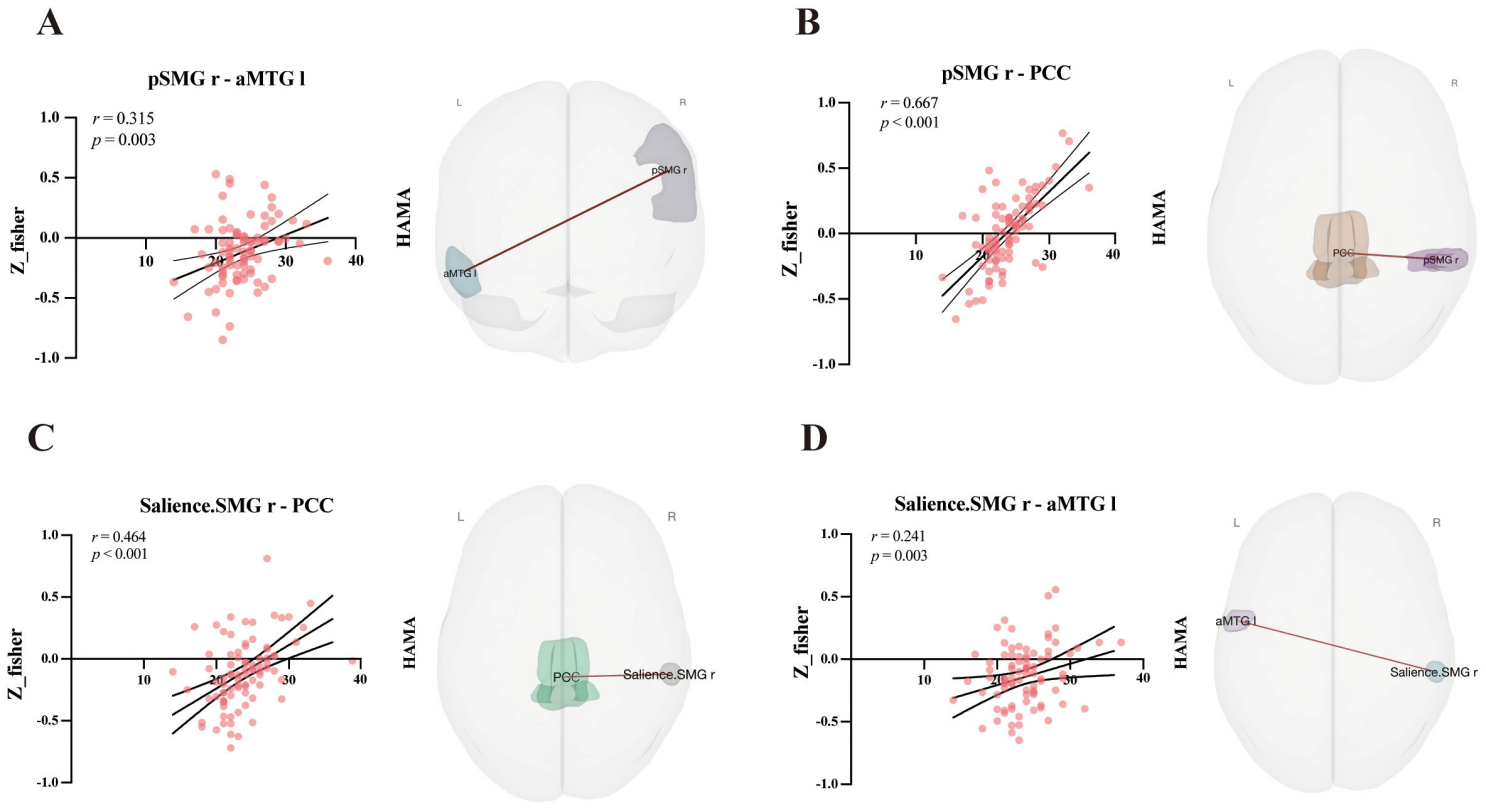
Displays significant correlations (Pearson’s r) between HAMA scores and FC values for four ROI - ROI pairs, with Subgraphs A–D respectively illustrating: **(A)** Correlation of pSMG r–aMTG l (posterior Supramarginal Gyrus right – anterior Middle Temporal Gyrus left). **(B)** Correlation of pSMG r–PCC (posterior Supramarginal Gyrus right – Posterior Cingulate Cortex). **(C)** Correlation of Salience.SMG r–PCC (Salience Network - Supramarginal Gyrus right – Posterior Cingulate Cortex). **(D)** Correlation of Salience.SMG r–aMTG l (Salience Network - Supramarginal Gyrus right – anterior Middle Temporal Gyrus left). Adjacent 3D brain renderings (posterior view of brain; L = left, R = right), generated using CONN toolbox, visualize the spatial locations of relevant brain regions and their connectivity pathways.

Moderate correlations were identified between HAMA scores and FC (**Table 3**). Specifically, positive correlations were found for the following connections: pSMG r – aMTG l; pSMG r – PCC; Salience.SMG r – PCC; Salience.SMG r – aMTG l. The correlation analysis and its brain connections are shown in **Figure 2**.

**Table 3:** Significant correlations between FC and clinical scores.

Scale	ROI	ROI	*r*	*P*
HAMA	pSMG r	PCC	0.667	< 0.001
	pSMG r	aMTG l	0.315	0.003
	Salience.SMG r	PCC	0.464	< 0.001
	Salience.SMG r	aMTG l	0.241	0.003

*r* = Pearson correlation coefficient. HAMA scores and FC z-scores met normality assumptions via Shapiro-Wilk test; associations were evaluated using Pearson correlation with *p* < 0.05 (uncorrected for multiple comparisons). pSMG r and Salience.SMG r show partial spatial overlap, with Salience.SMG r covering a broader area and pSMG r limited to an SMG r subregion (l, left; r, right).

### 3.4 Seed-based connectivity results

**Figure 3:**
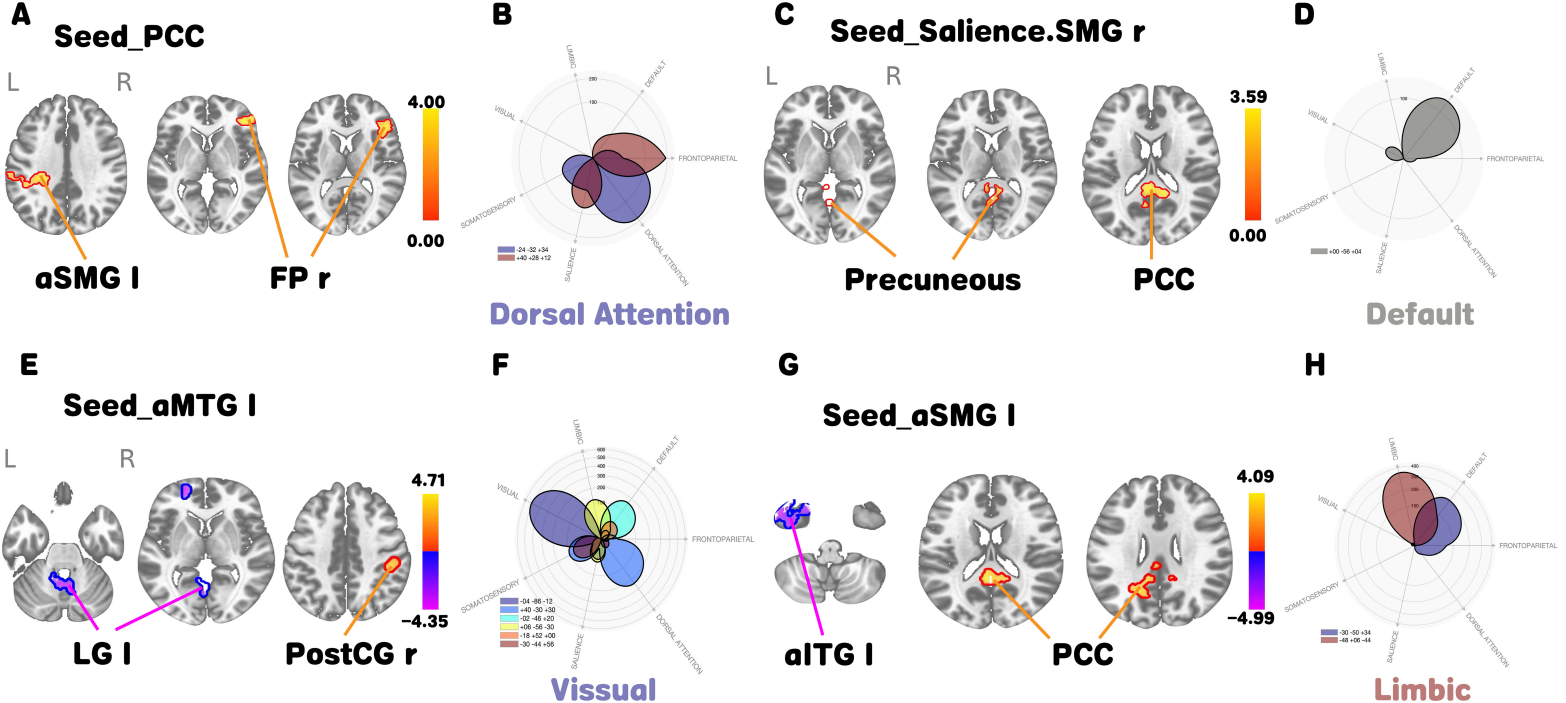
**(A)** Brain regions with increased whole-brain FC using the PCC as seed. **(B)** Network patterns via CONN Polar Display, with key networks labeled below. **(C–H)** Interpreted similarly.

Seed-based connectivity findings are presented in **Figure 3**. With the PCC as seed, increased rsFC was noted with the right SMG and right prefrontal regions. For the right SMG in the salience network (Salience.SMG r) as seed, increased rsFC occurred in the PCC and precuneus. The left anterior middle temporal gyrus (aMTG l) seed showed decreased rsFC with the left lingual gyrus and increased rsFC with the right postcentral gyrus. The left SMG seed revealed decreased rsFC with the left anterior inferior temporal gyrus (aITG l) and increased rsFC with the PCC.

## 4 Discussion

This study reports novel enhanced rsFC between the right SMG and PCC in medication-naïve patients with generalized anxiety disorder (GAD). This connectivity correlated strongly with Hamilton Anxiety Rating Scale (HAMA) scores (*r* = 0.667, *p* < 0.001), implicating SMG - PCC interactions in GAD pathophysiology.

ROI-to-ROI analyses identified increased right SMG-PCC FC, while seed-to-voxel analyses with the PCC as seed revealed further enhancements involving the left anterior SMG and right PFC. The SMG contributes to sensory integration, working memory, and executive control (38). Prior GAD studies indicate SMG abnormalities, such as reduced right SMG-superior parietal gyrus rsFC (39) and elevated local gyrification index in the SMG (40), a marker of psychiatric risk (41). Heightened SMG activity in GAD may result in misattribution of neutral stimuli as threats, intensifying hypervigilance.

The right SMG volume associates with emotion recognition (42), and the SMG supports overcoming emotional egocentricity (43) and mental state inference (44). Increased right SMG-PCC-medial prefrontal cortex (mPFC) FC could signify amplified SMG engagement in anxiety, modulating self-referential and socio-emotional processes through PCC interoception and mPFC cognition. Hemispheric asymmetry is evident: the left SMG focuses on tool use and language (45, 46), whereas our data emphasize right SMG dominance in GAD.

Midline default mode network (DMN) hubs, including the PCC and anterior mPFC, engage in self-referential emotional processing (47). Salience network (SN) -DMN dysconnectivity correlates with anxiety (48), with reduced intra-DMN rsFC involving the PCC and SMG in high-anxiety states (49) and dynamic limbic-prefrontal/DMN decrements in GAD (50). Our observed right SMG-PCC-mPFC hyperconnectivity suggests dysregulated network coupling, fostering threat misinterpretation and sustained vigilance.

Current models posit GAD anxiety stems from disruptions beyond acute fear circuits, encompassing sustained threat, reward, cognitive control, and socio-emotional domains (51). High anxiety involves intensified negative emotion processing, disrupting regulation (52). The right SMG, key for interoceptive awareness and attention, may exaggerate bodily (e.g., heartbeat) or threat signals in GAD, driving hypervigilance (53). The PCC, a DMN core (54, 55), sustains self-referential negative focus, fueling worry cycles. Right SMG-PCC hyperconnectivity may indicate DMN-fronto-parietal network (FPN) misalignment (56), excessive integration of interoceptive/threat cues at rest and amplifying symptoms. These findings are consistent with an interoceptive bias in GAD, where bodily signal hypersensitivity sustains anxiety; in resting-state fMRI, such rsFC may reflect threat attribution to benign cues absent external stimuli.

These findings on enhanced PCC-SMG connectivity could serve as a potential biomarker for identifying GAD subtypes, enabling personalized interventions such as fMRI-guided neurofeedback to normalize interoceptive networks and reduce anxiety symptoms. Furthermore, targeting interoceptive hypersensitivity through therapies like mindfulness-based stress reduction or interoceptive exposure may improve clinical outcomes in medication-naïve patients, bridging neuroimaging insights to real-world treatment strategies.

A key limitation of this study is the absence of direct measures of interoceptive awareness and attention, which constrains precise inferences about the relationship between supramarginal gyrus (SMG) activation and subjective bodily alertness. Although fMRI data highlight SMG’s pivotal role in the somatosensory awareness network (57), and its potential recruitment of the posterior cingulate cortex (PCC) and medial prefrontal cortex (mPFC) in emotional processing (58), these findings remain correlational without behavioral or physiological validation (e.g., heartbeat detection tasks). Future research should incorporate multimodal approaches, such as interoceptive sensitivity scales and heart rate variability monitoring, to confirm SMG-driven vigilance in amplifying anxiety symptoms (59). Moreover, longitudinal designs could elucidate the dynamic recruitment of PCC/mPFC in precipitating anxiety cycles over time (60), informing targeted interventions like interoception-focused cognitive behavioral therapy.

No alterations emerged in typical GAD regions like the amygdala (61, 62), hippocampus (63), ACC (64), or dorsomedial PFC (65). This may arise from our data-driven whole-brain method and exclusive medication-naïve sample, yielding distinct cohort features versus prior work.

The GAD sample showed gender imbalance (60 females, 25 males), covaried in analyses. GAD prevalence is twice as high in females (66), with sex influencing anxiety neurobiology (67–69). Future studies must probe sex effects. Seed-based connectivity power may be limited (70), and broader sample heterogeneity could constrain generalizability (71). Clinically, SMG-PCC rsFC could target interventions like transcranial magnetic stimulation or interoception-focused therapies.

A further limitation concerns illness duration. Although this variable was collected, it was not entered as a covariate in the primary group-level rs-fMRI analyses. In functional connectivity studies of GAD, disease duration has generally shown weaker and less consistent effects compared with structural MRI studies (e.g., gray matter volume loss or cortical thickness reduction). Moreover, self-reported duration in GAD patients is often imprecise and highly variable because of the insidious onset and fluctuating course of symptoms, which reduces its reliability as a quantitative covariate. For these reasons, and consistent with many prior rs-fMRI investigations of anxiety disorders (72–74), we controlled only for age and gender in the main models.

## 5 Conclusions

In this study, patients with GAD exhibited enhanced rsFC between the posterior cingulate gyrus and the SMG r, which was highly positively correlated with the HAMA scale scores. These findings suggest a potential role for heightened SMG activity in contributing to interoceptive vigilance, potentially engaging the PCC and mPFC in emotional processing that may perpetuate anxiety cycles; however, direct measures of interoceptive awareness (e.g., heartbeat detection tasks) are required to validate this mechanism in future research. This pattern underscores interoceptive hypersensitivity as a potential core feature of GAD, warranting further exploration into its etiological contributions.Interoceptive sensitivity (75, 76), plays a crucial role in anxiety, and therapeutic approaches targeting interoceptive regulation may become important targets for the treatment of GAD.

## Data Availability

The raw data supporting the conclusions of this article will be made available by the authors, without undue reservation.
